# Acquired Long QT Syndrome and Torsade de Pointes Associated with HIV Infection

**DOI:** 10.1155/2010/278427

**Published:** 2010-08-23

**Authors:** Alexander Shimabukuro-Vornhagen, Jan Rybniker, Shahram Zoghi, Gerd Faetkenheuer, Guido Michels, Erland Erdmann, Michael von Bergwelt-Baildon, Matthias Kochanek

**Affiliations:** ^1^Department I of Internal Medicine, University Hospital of Cologne, 50937 Cologne, Germany; ^2^Department III of Internal Medicine, University Hospital of Cologne, 50937 Cologne, Germany

## Abstract

Here, we report the case of an HIV infected patient that was treated for pneumonia with a macrolid antibiotic. The patient experienced a prolongation of the already pathologic QTc interval resulting in repeated torsades de pointes necessitating CPR and implantation of an AICD. This case exemplifies that torsades de pointes due to acquired long QT syndrome is a serious and potentially fatal complication in HIV-positive patients.

Human immunodeficiency virus- (HIV-) infected individuals are at an increased risk of cardiovascular disease [1, 2]. Here, we report the case of an HIV-infected patient with acquired long QT syndrome and multiple episodes of torsades de pointes necessitating repeated cardiopulmonary resuscitation. A 41-year-old HIV-positive patient presented with dyspnea, cough, and fever to our emergency department. The patient had just returned from a two-week vacation to Egypt. In 1997, he was diagnosed with HIV and an antiretroviral therapy (ART) was started. The patient discontinued the ART in 2004 and at the time of admission he was not taking any medications. He, furthermore, complained of HIV-associated neuropathy of the hands and feet. Chest X-ray revealed bilateral infiltrates, a diagnosis of bacterial pneumonia was made and an inpatient treatment with ceftriaxone and clarithromycin was initiated (CD4 count 0.06x1E9/l).

Two days after admission the patient experienced a syncope. Since there was no palpable pulse, cardiopulmonary resuscitation was performed by the medical team for about 30 seconds after which the patient had regained consciousness. The patient was transferred to the medical intensive care unit for further observation. There was no prior history of syncope, seizures, or cardiac events. The 12-lead electrocardiogram showed QT prolongation with a corrected QT interval (QTc, Bazett's correction) of 660 ms. In the following hours the patient had repeated episodes of polymorphic ventricular tachycardias that were either self-limiting or required defibrillation. Figure 1 shows representative ECGs recorded during some of these episodes. Between the episodes, the patient's ECG was characterized by bradyarrhythmia with intermittent complex ventricular premature complexes with bursts of couplets (Figure 1(a)). A premature ventricular beat (Figure 1(b)) precipitated polymorphic ventricular tachycardias with the typical electrocardiographic features of torsades de pointes (Figure 1(c)). Following administration of mexiletine hydrochloride, no further ventricular tachycardias occurred. Due to the high likelihood of future treatment with QT-prolonging drugs and the high risk of repeated events, the patient received an automated implantable cardioverter defibrillator (AICD). During the follow-up no further cardiac events were reported.

Long QT syndrome is more frequent in subjects infected with HIV than in the general population and has been reported to occur in up to 29% of the hospitalized HIV-positive patients [2, 3]. Why long QT syndrome occurs more often in HIV-positive patients is currently unknown. There are multiple possible explanations why long QT syndrome is more prevalent in HIV-infected patients, though. HIV-positive patients frequently receive medications that prolong the QT interval such as diflucan, clarithomycin, and cotrimoxazol. Furthermore, antiretroviral drugs themselves have been implied to cause prolongation of the QT interval [4–6]. Table 1 summarizes drugs that are frequently given to patients with HIV and that are thought to be associated with QT prolongation. Whether HIV infection itself might cause heart disease and QT-interval prolongation is controversial. In our patient, medications alone do not explain the presence of long QT syndrome since review of the ECG obtained upon admission already showed prolongation of the QT interval with a QTc of 474 msec. Thus, other mechanisms must have contributed to the prolongation of the QT interval. Autonomic dysfunction due to HIV-associated neuropathy is another presumed cause of long QT syndrome in HIV patients and could have been a contributing factor in our patient, who suffered from HIV-associated peripheral neuropathy [7, 8].

This case exemplifies that torsades de pointes due to acquired long QT syndrome is a serious and potentially fatal complication in HIV-positive patients. Multiple factors including antimicrobial drugs put HIV-infected patients at an increased risk for the development of acquired long QT syndrome. Physicians should therefore always maintain a high degree of clinical suspicion for the presence of long QT syndrome in patients with HIV and should be aware of the QT-prolonging side effects of drugs they prescribe for these patients.

## Figures and Tables

**Figure 1:**
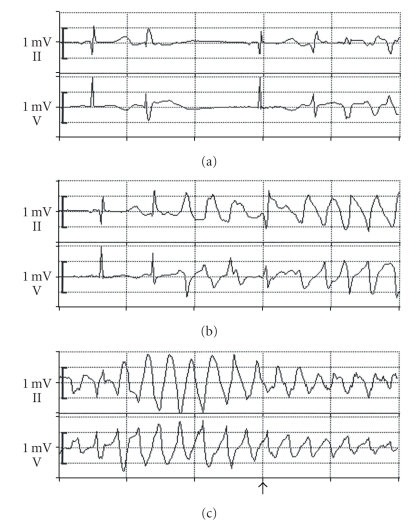


**Table 1:** 

*Antibiotics*
Clarithromycin
Erythromycin
Pentamidine
Levofloxacin
Moxifloxacin
Ciprofloxacin
Cotrimoxazole (trimethoprim-sulfamethoxazole)

*Antifungals*
Voriconazole
Fluconazole
Itraconazole

*Antiretrovirals*
Atazanavir
Efavirenz
